# Autopsies Revealed Pathological Features of COVID-19 in Unvaccinated vs. Vaccinated Patients

**DOI:** 10.3390/biomedicines11020551

**Published:** 2023-02-14

**Authors:** Daniele Colombo, Franca Del Nonno, Luisa Marchioni, Eleonora Lalle, Paola Gallì, Francesco Vaia, Laura Falasca

**Affiliations:** 1 Pathology Unit, National Institute for Infectious Diseases “Lazzaro Spallanzani”-IRCCS, 00149 Rome, Italy; 2 Clinical Department, National Institute for Infectious Diseases “Lazzaro Spallanzani”-IRCCS, 00149 Rome, Italy; 3 Laboratory of Virology, National Institute for Infectious Diseases “Lazzaro Spallanzani”-IRCCS, 00149 Rome, Italy; 4 Health Direction, National Institute for Infectious Diseases “Lazzaro Spallanzani”-IRCCS, 00149 Rome, Italy; 5 Laboratory of Electron Microscopy, National Institute for Infectious Diseases “Lazzaro Spallanzani”-IRCCS, 00149 Rome, Italy

**Keywords:** autopsy, COVID-19, SARS-CoV-2 vaccine, pathology, lung, myocardium

## Abstract

**Background**: In Italy, by the end of 2021, a new pandemic wave led to increased hospitalizations and death, even in some vaccinated people. We aimed to investigate the death of COVID-19-vaccinated patients who acquired infection and developed severe disease, and to assess differences with fatal COVID-19 in unvaccinated subjects by studying the pathological events triggered by SARS-CoV-2. **Methods**: Detailed autoptic examination was performed on five fully vaccinated compared to five unvaccinated patients. Histopathological analysis focused on the lung and heart, the two major affected organs. **Results**: COVID-19 caused, or contributed to death, in all the unvaccinated cases. By contrast, in vaccinated group, pre-existing pathologies played a major role, and death was not COVID-19-related in four out of five patients. These patients did not show the histological features of SARS-CoV-2 lung damage. Diffuse inflammatory macrophages infiltration recently emerged as the main feature of COVID-19 cardiac injury. Interestingly, the most striking difference between the two groups was the absence of increased macrophage infiltration in the heart of vaccinated patients. **Conclusions**: Results of this study confirm the efficacy of anti-SARS-CoV-2 vaccination in protecting organs from injury and support the need to maintain an adequate immune response by booster dose administration.

## 1. Introduction

Severe COVID-19 has a high mortality rate due to occurrence of multiple organ dysfunction syndrome [1,2]. Although underlying co-morbidities and old age represent major risk factors for fatal progression, a number of COVID-19 deaths have occurred in young and previously healthy individuals [3,4].

Given the lack of specific treatment, vaccination against SARS-CoV-2 was considered the most promising strategy to end the COVID-19 pandemic. Different vaccines were developed globally and mRNA vaccines, authorized first for emergency, proven their safety and efficacy [5,6]. Optimal protection was gained with a full vaccination protocol of two doses, with an interval of three weeks between them [7]. The immune response, elicited in vaccinated individuals, has demonstrated to reduce risk of critical hospitalization and deaths associated with severe COVID-19 [8,9,10]. Nevertheless, the duration of vaccine efficacy decreases with time, waning 4–6 months after the full vaccination, especially in older people [11]. The purpose of this study was to shed light on the causes of deaths of SARS-CoV-2-vaccinated patients who developed severe COVID-19. Patient enrollment took place between October 2021 and February 2022.

The start of the vaccination campaign in Italy was on 27 December 2020, according to the authorized use of the vaccine BNT162b2 (Pfizer–BioNTech) provided by the European Union on 21 December 2020 [12]. On February 2022, in Italy, there were 77.88% fully vaccinated people and a daily death rate, due to COVID-19, of around 320. In that time, the mortality rate of unvaccinated people was about six times higher than individuals vaccinated with a full course (for less than 120 days) [13].

The aim was to assess similarities and differences with fatal COVID-19 cases of unvaccinated patients, with a focus on lung and heart involvement. We present a detailed autopsy study of five positive SARS-CoV-2 patients who were double-dose vaccinated (fully vaccinated) against SARS-CoV-2, compared to five unvaccinated patients (subjects who had not yet received any vaccine dose of any kind). The study was undertaken during the Delta and Omicron variants.

## 2. Materials and Methods

### 2.1. Study Cohort

Cases included in this study were 10 COVID-19 patients who underwent complete postmortem examination at National Institute for Infectious Diseases “Lazzaro Spallanzani”-IRCCS Hospital in Rome, Italy, between October 2021 and February 2022. Cases considered for the study included five consecutive vaccinated and five unvaccinated patients. All patients enrolled had resulted positive for COVID-19, tested (either before and/or at hospital admission) by nasopharyngeal swab. Other inclusion criteria were as follows: age > 18 years old, presence of symptomatic conditions (respiratory, clinical, and/or radio-logical picture suggestive of COVID-19), and hospital attendance (including admissions and attendances at emergency departments). Vaccinated patients were individuals classified as fully vaccinated, since they had completed the vaccination course (double doses) of the Pfizer-BioNtech vaccine.

The study was approved by the Institutional Ethic Board (IEB) of the National Institute for Infectious Diseases “L. Spallanzani” (approval number: 3/2022). All data were anonymized to protect the confidentiality of individual participants.

### 2.2. Autopsy Procedures

Autopsies were carried out by a team of highly trained personnel in a negative pressure biosafety level 3 (BSL-3) room. Complete postmortem examinations were performed. Organs were removed and macroscopically inspected. Brain harvesting was performed in only 6 patients (3 vaccinated and 3 non vaccinated) whose clinical history reported neurological problems or pathologies. The lungs were weighed, and multiple lung samples from each pulmonary lobe (upper right, middle right, lower right, upper left, lower left) were taken. The heart was weighed, and then cut transversely from the apex to mid ventricle at 1 cm intervals; the remaining heart was opened following the lines of blood flow. Specific heart parameters were measured: thickness of the right ventricle wall, left ventricle wall, and septum wall. Multiple samplings were created for the histology.

### 2.3. SARS-CoV-2 RT-PCR

All patients were diagnosed as COVID-19-positive by PCR test for SARS-CoV-2 performed on nasopharyngeal swab and/or on autoptic samples. Postmortem swabs from different organs were pre-treated with 1:1 ATL lysis buffer, and total nucleic acids (DNA/RNA) were extracted using the QIAsymphony^®^ instrument with QIAsymphony^®^ DSP Virus/Pathogen Midi Kit (Complex 400 protocol) according to the manufacturer’s instructions (QIAGEN, Qiagen, Hilden, Germany). SARS-CoV-2 qualitative reverse-transcriptase–polymerase chain reaction (RT-PCR) testing using RealStar^®^ SARS-CoV-2 RT-PCR Kit 1.0 targeting E and S genes (Altona Diagnostic GmbH, Hamburg, Germany) was performed according to the manufacturer’s instructions, on the QIAGEN Rotor-Gene Q 5 PLEX platform. The number of cycles (Ct values) needed to detect the viral RNA was considered to better understand the relative amounts of genetic material present in the samples (the lower the Ct value, the more viral RNA was in the sample). The threshold of Ct value under which a sample was interpreted as positive was 40.

### 2.4. Histological Anlysis

Organ samples were immediately fixed in neutral-buffered formalin and stored for 72 h before inclusion into paraffin blocks. For histopathological analysis hematoxylin and eosin (H & E)-stained tissue sections (4 μm) were used. Tissue analysis and images of the slides were obtained with a Leica DM 2500 Led (Leica Microsystems, Mannheim, Germany) light microscope, equipped with a Leica MC190 HD camera. The severity of lung injury was established considering different histological parameters, analyzed at magnifications of 40×, and scored according to the percentage of tissue area involved. The following features were considered: hyaline membranes, alveolar and interstitial inflammation, alveolar hemorrhage, microthrombi, alveolar and interstitial fibrosis, remodeling, and edema. A total score was calculated for each patient as mean of 10 slides of lung tissue.

Evaluation of myocardial lesions was based on degree of interstitial fibrosis, vascular fibrosis, myocarditis, inflammatory infiltrate, myocyte hypertrophy, and myocyte disarray. The extent of these histological parameters was quantified on the basis of the area of tissue involved. A total score was calculated, for each patient, as mean of 5 slides of cardiac tissue.

### 2.5. Immunohistochemistry

Deparaffinized and rehydrated sections from the lung and heart were used for immunohistochemistry. Immunostaining was done with BenchMark Ultra IHC/ISH System fully automated instrument (Roche, Basel, Switzerland), using the ultraView Universal diaminobenzidine (DAB) Detection Kit (760–500, Roche-Ventana, Tucson, AZ 85755, USA), in accordance with the standard protocols supplied by the manufacturer. All antibodies were ready-to-use monoclonal antibodies (Ventana, Roche Diagnostics, Basel, Switzerland); antibodies directed against CD3 (Ventana, 2GV6) CD4 (Ventana, SP35), CD8 (Ventana SP57), CD20 (Ventana, LZ6), CD68 (Ventana KP-1), and CD45 (Ventana, LCA) were used. Negative control stainings were performed by omitting the primary antibodies.

All cases were independently analyzed by two pathologists without knowledge of the clinical diagnosis. The extent of immunoreactivity was assessed by using the same microscope at a magnification of 40×. The number of positive cells was quantified and expressed as cells/mm^2^.

### 2.6. Statistical Analyses

Data were reported as mean or median as appropriate. Discrete parameters were expressed as counts or percentages. Data were analyzed using Prism Software (GraphPad, version 5). Statistical significance was analyzed by a two-sided, unpaired Student’s *t* test. A *p* value < 0.05 was considered statistically significant.

## 3. Results

### 3.1. Patients Demographics and Clinical Characteristics

Patients enrolled for this study were White, non-Hispanic. All subjects attended to a hospital with symptomatic conditions and were positive for the COVID-19 nasopharyngeal swab test. The demographics, medical history, clinical features, and postmortem diagnosis of our cohort of patients are summarized in Table 1 and Table 2. The five vaccinated patients were subjects who had completed the primary series of vaccination, with two doses of the BNT162b2 Pfizer-BioNTech vaccine (Table 1).

All of them received the second dose more than 6 months before SARS-CoV-2 infection, with a median time between vaccination and death of 8.5 months (ranging from 6.5 to 10 months). All cases had medical co-morbidities, with three patients suffering for more than three pre-existing pathologies. Cerebral diseases, cancer, rheumatoid arthritis, and heart disease were frequent conditions. In this group of patients, there were two females and three males, with a median age of 75 years (range 60–81 years) (Table 1). No one of these persons lived in assisted retirement facilities or in long-term care facilities. A significant difference (*p* ≤ 0.05) was observed concerning the age of patients in the two groups, with the unvaccinated group being younger, presenting a median age of 51 years (age ranged between 45 and 67 years) (Table 2).

Among this group, which included two males and three females, only two patients showed chronic diseases, two were considered obese (body mass index [BMI] ≥ 30), and one subject presented no underlying medical conditions (Table 2).

### 3.2. Cause of Death

According to the WHO guidelines for certification of death [14], four out of five unvaccinated patients died with COVID-19 acute respiratory distress syndrome, as ascertained by autopsy, and two of them with associated myocarditis. Regarding the remaining case (case n. 3), COVID-19 represented a condition contributing to death, which occurred for massive gastrointestinal hemorrhage (Table 2). In the vaccinated group, the cause of death was acute respiratory distress syndrome due to COVID-19 in only one case (case n. 1). The other four patients died from pre-existing conditions not related to COVID-19.

### 3.3. Postmortem SARS-CoV-2 PCR Analysis

The presence of viral RNA was evaluated postmortem, by RT-PCR analysis, on autoptic swabs from different organs (Table S1). Viral RNA was found in bronchi and lungs from all patients, unvaccinated as well as vaccinated subjects; myocardium displayed positive results in two vaccinated and four unvaccinated patients (Table S1). The level of viral load was deduced from the Cycle threshold (Ct) value, being the lower the Ct value, the higher the viral load. Interestingly, the median value of viral load showed a significant higher presence (lower Ct values) in lungs of unvaccinated patients compared to vaccinated group (21.49 vs. 30.54, *p* ≤ 0.005) (Table 3). 

In bronchi and myocardium, the amount of SARS-CoV-2 RNA did not reach significant differences (Table 3).

### 3.4. Autopsy and Microscopic Examination

#### 3.4.1. Lung Findings

The gross findings of the lungs are summarized in Table 4. All the unvaccinated patients (except case 1, which was emphysematous) presented a combined right and left lung weight greater than the upper limit for normal lung weight (1300 g) [15,16], with a median weight of 1793 g (Figure S1). In the majority of cases, both the right and left lung weights were greater than the reference ranges (right lung: 155–720 g; left lung: 112–675 g) [17]. Among vaccinated patients, the median of the combined weight was 1146 g (Figure S1), with only two cases presenting an increased lung weight (Table 4).

Table 1 and Table 2 and macroscopic examination (Table 4). Diffuse consolidation of all lobes was present in four out of five unvaccinated patients. On the contrary, in vaccinated subjects (except case n.1), consolidation was focally distributed or even absent in the different lobes, with a diffuse pattern when associated with bacterial bronchopneumonia (Table 4). Edema, congestion (with deep red, firm and rubbery cut surface), and different degrees of areas of gray fibrous tissue were macroscopically found in both groups of patients. Pulmonary embolism was observed in a single case, among vaccinated patients, while in only one unvaccinated patient, artery thrombosis was seen.

**Figure 1:**
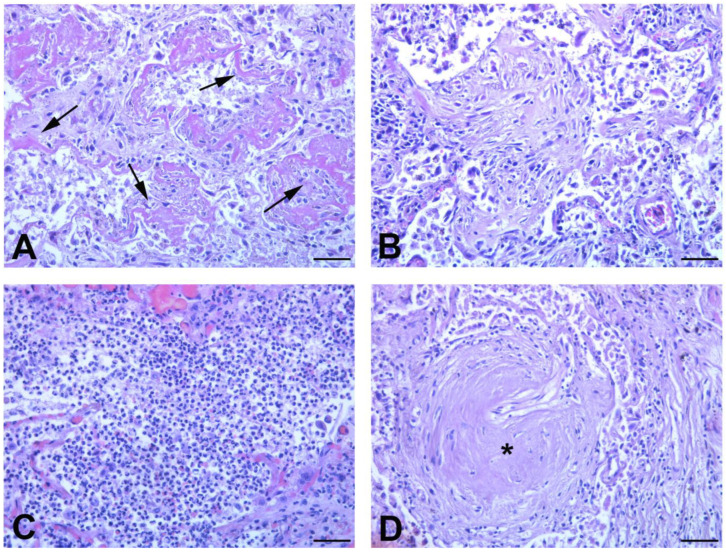
Histopathological features of autoptic lung from unvaccinated and vaccinated COVID-19 patients. (**A**,**B**) Representative Hematoxylin and Eosin-stained section, from lung tissue of unvaccinated patients. (**A**) Acute exudative pattern of diffuse alveolar damage (DAD), with hyaline membranes deposition (arrows). (**B**) Intra-alveolar plug of organizing connective tissue (proliferative/organizing phase). (**C**,**D**) Representative histological findings in lung tissue of vaccinated patients: (**C**) massive neutrophils infiltration indicative of bacterial bronchopneumonia; (**D**) arthritis rheumatoid fibrotic nodule (*). Scale bars: (**A**–**D**) = 28 μm.

Light microscopy examination of lung tissue revealed bilateral diffuse alveolar damage (DAD) and acute (exudative phase) (Figure 1A) and organizing patterns (Figure 1B) in all the four unvaccinated subjects who died from COVID-19.

In contrast, in vaccinated group, the acute exudative phase of DAD was present focally in just one patient (case n.1), while architectural remodeling, associated with the zone of active fibrosis, was found in the lung of all subjects. Other histological features in these patients were bacterial bronchopneumonia (Figure 1C) and fibrotic nodules, which are specific hallmarks of rheumatoid arthritis (Figure 1D). 

**Figure 2:**
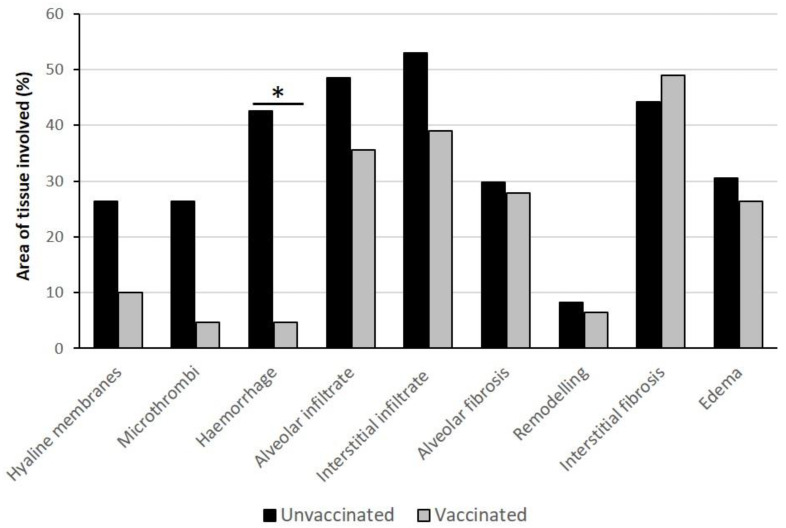
**Histology score of lung damage in unvaccinated and vaccinated COVID-19 patients.** Histological features of lung damage were scored according to the percentage of the tissue area involved. Some pathological changes show higher values in unvaccinated vs. vaccinated patients. * Significant difference: *p* ≤ 0.01.

Assessment of lung injury, based on the histology scoring system, showed a trend to a more severe presence of hyaline membranes, microthrombi, and inflammatory infiltrate in unvaccinated patients (Figure 2) and a significantly higher score concerning the extension of hemorrhage (*p* ≤ 0.01) compared to vaccinated lungs.

The extension of hyaline membranes and the presence of microthrombi (which are recognized features of COVID-19 pneumonia) did not reach statistical significance because one case (case n.1), among vaccinated patients, had an acute lung injury due to COVID-19. No differences were observed for fibrosis, but this result was not surprising, considering the underlying pathologies of vaccinated patients, such as Parkinson’s disease and rheumatoid arthritis, which are recognized causes of pulmonary fibrosis. As concerns inflammatory cells, massive neutrophils infiltration was found in areas affected by bacterial pneumonia (four vaccinated patients, and one unvaccinated patient), while macrophages (CD68+) and lymphocytes (CD3+) were always observed. The results of immunohistochemical analysis showed a variable number of CD68+ cells (range: 39.00–131.33/mm^2^ in unvaccinated, vs 38.33–141.67/mm^2^ in vaccinated patients) and CD3+ T lymphocytes (range: 47.50–372.67/mm^2^ in unvaccinated patients, vs. 17.00–141.33/mm^2^ in vaccinated patients) (Figure S2). No statistically significant difference emerged between the two groups, as expected, due to underlying pathologies in vaccinated patients, which may cause chronic recruitment of inflammatory cells.

#### 3.4.2. Heart Findings

Macroscopic examination of heart revealed that the weight of the organ in unvaccinated patients presented a range from 396 to 797 g, with a median weight of 436 g (Table 5).

**Figure 3:**
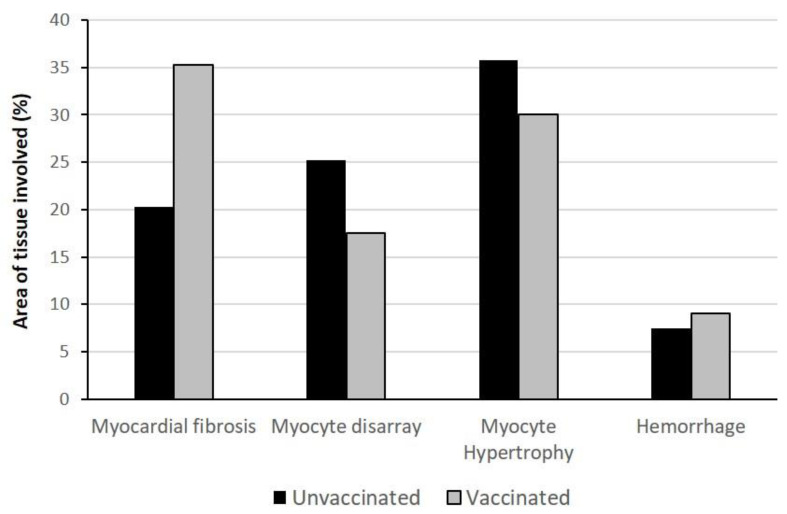
**Histology score of heart damage in unvaccinated and vaccinated COVID-19 patients.** Histological score of myocardial injury determined by the percentage of the tissue area involved. A trend towards more severe fibrotic damage is present in vaccinated patients.

In vaccinated patients, the median heart weight was 427 g, presenting a range of 332–470 g (Table 5) (Figure S3). In this group, cardiac hypertrophy, chambers dilation, fibrosis, and atherosclerosis, complicated by calcification of mitral annulus and aortic valve and/or coronaries stenosis, were the main pathological findings, which appeared to be associated with the older age of these patients and to the presence of chronic comorbidities. In the unvaccinated patients, four cases showed cardiac hypertrophy, which was associated with ventricular dilatation in only two cases (Table 5). Microscopic examination of heart sections showed the presence of myocardial fibrosis, myocytes disarray, myocytes hypertrophy, and micro-hemorrhagic areas. Histological scores revealed a variable degree of the extent of these pathological changes in both vaccinated and unvaccinated patients (Figure 3); however, the presence of underlying pathologies made the interpretation of these signs of cardiac injury difficult in the vaccinated cohort of patients.

**Figure 4:**
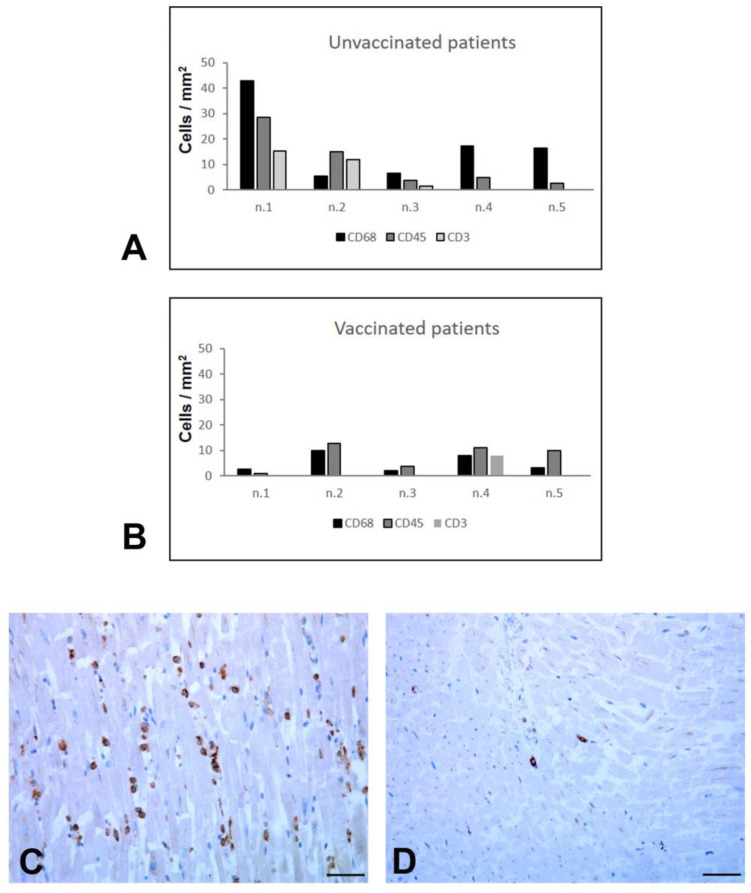
Inflammatory cells immunemcharacterization in myocardial tissue. (**A**,**B**) Quantification of the number of extravascular CD68+ macrophages, CD45+ leukocytes, and CD3+ T lymphocytes. Unvaccinated and vaccinated patients display a different pattern of inflammatory cells. For each case (n.1–n.5) the mean number of cells per mm^2^ is reported. (**C**,**D**) Representative images of anti-CD68 immunohistochemistry. (**C**) The immunolabelling of myocardial tissue from unvaccinated patient highlights the presence of numerous macrophages. (**D**) Rare CD8-positive cells are visible in myocardial tissue from vaccinated patient. Scale bars: (**C**,**D**) = 28 μm.

By contrast, as regards the inflammatory changes, a different pattern between the two groups was found (Figure 4A,B).

**Figure 5:**
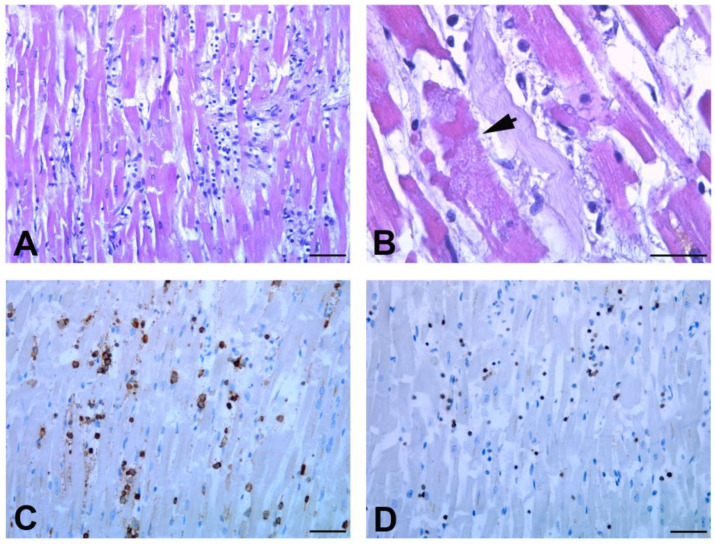
Histopathological feature of myocarditis in unvaccinated patients. (**A**,**B**) Hematoxylin and Eosin-stained sections of myocardial tissue show multifocal myocarditis, with (**A**) patchy areas of inflammatory cell infiltrates associated with ((**B**), arrow) myocyte necrosis. (**C**,**D**) Immunophenotypic characterization of inflammatory cells, identify the presence of (**C**) CD45+ leukocytes and (**D**) CD3+ T lymphocytes, according to definition of myocarditis. Scale bars: (**A**,**C**,**D**) = 28 µm; (**B**)= 30 µm.

Immune histochemical characterization showed, in the myocardium of all unvaccinated patients (except case 2), a diffuse increased number of interstitial macrophages (CD68+) (Figure 4C). On the contrary, macrophages were mostly rare in vaccinated subjects (Figure 4D). It is noteworthy that, in two cases (n.1 and n.2) of the unvaccinated group, multifocal lymphocytic myocarditis was also seen (Figure 5), characterized by predominantly lymphocytic infiltrate associated with myocyte necrosis according to the Dallas Criteria (more than 14 leukocytes/mm^2^, with >7 T lymphocytes/mm^2^) and to the subdivision of Basso et al. [18].

One patient (n.4) among the vaccinated group displayed feature of myocarditis (Figure 4B) that was most likely associated with cirrhotic cardiomyopathy, as supported by the alcoholic encephalopathy highlighted at the clinical and histological levels [19]; one other patient (n.2) showed myocardial infiltration of CD68- and CD45-positive cells for acute and chronic ischemic cardiomyopathy (Figure 4B).

#### 3.4.3. Other Organs

Tissue autolysis did not permit detailed histological analysis of the gastrointestinal tract in most cases. The histological changes found in the liver and kidneys could be related to pre-existing comorbidities. Only one unvaccinated patient (n.1) showed histological features of secondary sclerosing cholangitis, which is probably associated with COVID-19 [20]. Brain examination was performed on six patients: patients n.1, n.4, and n.5 of the vaccinated group and patients n.1, n.2, and n.3 of the unvaccinated group. Neuropathological findings are summarized in Table S2. Histopathological features of hypoxic-ischemic damage, such as axonal vacuolization, degenerations of neurons, and reactive gliosis, were observed in one patient (n.1) in the unvaccinated group and in one patient (n.5) with a history of epilepsy and cerebral vasculopathy in the vaccinated group. Two patients (n.1 and n.2) in the unvaccinated group showed vascular changes and perivascular lymphocytic inflammation of predominantly CD8-positive T cells. These findings were in line with neuropathological features previously described in autoptic COVID-19 brains [21]. The other cases showed specific histological features related to their neurological disorder as reported in Table S2 (patient n.3 of unvaccinated; patient n.1 and n.4 of vaccinated).

## 4. Discussion

Starting from October 2021, the increase of severe cases led Italy to a new emergency. Most of patients hospitalized with COVID-19 were unvaccinated. At that time, the unvaccinated Italian people amounted to 8 million, and the age group with the higher number of unvaccinated people, in absolute terms, was 40–49 years, followed by 50–59 years [22]. In the same period, the age groups 80+ and 70–79 achieved the highest vaccination coverage (93.6% and 91.1% of second doses, respectively), but the spread of more contagious SARS-CoV-2 variants highlighted the waning over time of the vaccine protection, especially in elderly and immunocompromised subjects. Consistent with these data, in our study, patients belonging to the unvaccinated group had a median age of 51 years, while the vaccinated patients were significantly older, with a median age of 75 years. Despite that vaccinated patients were older and had several pre-existing pathologies, they did not show the lung histological changes typically associated with COVID-19 injury [4,23]. Unvaccinated patients presented acute diffuse alveolar damage (DAD), marked hemorrhage, and organizing phase of DAD. In addition, more severe microvascular damage, characterized by platelet-fibrin thrombi in microvessels, was present. Unvaccinated patients also showed a significant higher lung viral load, compared to vaccinated group, as demonstrated by the low Ct values detected by RT-PCR. In this agreement was also the clinical manifestation of 60% of unvaccinated patients, who presented dyspnea, a clear symptom of lung damage.

We focused our attention on the heart, since this organ is often affected in severe COVID-19 [24], and an interesting difference emerged between the two groups in regards to inflammation. Other authors have described the presence of infiltrating inflammatory cells in the heart of deceased COVID-19 patients, and a peculiar inflammatory pattern emerged, in which an increased number of interstitial macrophages represented the main feature [25,26]. Our findings in the myocardium of unvaccinated patients confirmed that a diffuse number of CD68-positive macrophages were present in the interstitium, with a proportion ranging from 68 to 100%, compared to CD3+ lymphocytes. This diffuse inflammatory condition was not associated with myocyte necrosis. Probably, macrophages involvement does not induce a conventional myocyte damage, but is rather associated with fibrovascular interface remodeling, as highlighted by other authors [27]. 

By contrast, in the heart of the vaccinated patients, the increased macrophage infiltration was absent. This important finding appeared not related to the presence of virus, since viral RNA was detected in the heart of both groups. Considering the older age of vaccinated patients, we wondered whether this difference could be related to the so-called “immunosenescence”, a decline with aging of the immune response [28] that is thought to contribute to the pathogenesis of age-related diseases, in particular cardiovascular diseases [29,30]. In order to deepen this point, we evaluated the presence of inflammatory cells in myocardial tissue from five elderly patients (68–86 years; same range of vaccinated group), who were not vaccinated, extracted from our archives of COVID-19 autopsies. The results of this analysis (Figure S4) showed a number of CD45+ and CD3+ cells, and diffuse CD68+ macrophages distribution, resembling the inflammatory pattern found in the myocardium of unvaccinated group. These data provide further evidence that the difference in macrophages infiltration found in the heart of the vaccinated patients were actually due to vaccination and not to age. It could be speculated that, in the absence of vaccination, a worse lung damage may lead to a greater increase of systemic inflammatory mediators, which, in turn, could account for cardiac inflammation.

A still controversial topic concerns whether myocarditis is or not a manifestation of severe COVID-19 [31]. According to reports obtained from autoptic studies, the incidence of SARS-CoV-2-associated myocarditis is relatively low (1.4–7.2%). Apparently, in contrast with these data, here we showed two cases (40%) among the unvaccinated patients presenting histological features of multifocal myocarditis. Nevertheless, our results are in line with a study showing that COVID-19-associated myocarditis mainly affects young people [32]. The aim of this study was to address the question whether the deaths of vaccinated individuals who developed COVID-19 disease were attributable to SARS-CoV-2 infection. Among our cases series, four (80%) of the unvaccinated patients were listed as COVID-19-related deaths, while four (80%) of the vaccinated patients had the recognized cause of death listed for other causes. The interval period from the administration of vaccine is crucial since a reduction of the vaccine efficacy of 52% after 141–224 days has been established. The vaccinated patients we considered received the second dose more than 6.5 months before hospitalization. Of note, despite this long time interval, the majority of them (four out of five) became deceased from pre-existing pathologies. Consistent with data showing SARS-CoV-2 distribution in different tissues [33,34,35,36], we found, in some patients of both the vaccinated and unvaccinated groups, the presence of SARS-CoV-2 RNA in the kidneys, liver, and brain; however, the pathological modifications observed in these tissues appeared not to be correlated to a positive or negative RT-PCR test. In this agreement, electron microscopy investigations demonstrated that the presence of viral RNA and proteins does not necessarily correspond to the presence of virus particles [37]; thus, organ damage could be related to different pathological mechanisms occurring in COVID-19, as reported in the liver [38] and brain [39]. In this respect, autopsies provide an opportunity for clinicians and pathologists to learn more about various disease processes and to investigate how they manifest in the body. We believe that whole body autopsies offer several advantages, allowing for adequate tissue sampling for pathological and molecular evaluation and research. Postmortem swabs or tissue samples can be used to search infectious etiology with ancillary techniques such as PCR. During the COVID-19 pandemic, complete postmortem examination offered the only way to study the effect of COVID-19 disease on several organs and allow researchers to define the pathological alterations caused by SARS-CoV-2.

Our study has some limitations. First, the study is based on a small cohort of patients. Given the small number of deaths of SARS-CoV-2 fully vaccinated subjects with severe COVID-19, a limited number of autopsies of such cases was available. Second, the genotyping-confirmed variant information was available only in four cases (two patients for each group), all classified as Omicron lineage. We could estimate that the patients in which sequencing was not confirmed were very likely two Delta and one Omicron case for each group, according to the predominant circulating variant at the time of their infection. Despite these limitations, this study has the merit to add new information concerning the still remaining caveats of COVID-19 pathology. Most autoptic studies on COVID-19 regarded elderly patients, usually presenting a variety of underlying diseases, thus not allowing for a clear identification of SARS-CoV-2 pathological processes [40]. Here, we presented a pattern of lung and heart pathology in younger unvaccinated patients that could represent specific features of COVID-19 tissue damage. Future studies will be able to deepen the knowledge of our results.

## 5. Conclusions

In conclusion, our findings, identified by the invaluable tool of autopsy-based analysis of tissues, improve knowledge on SARS-CoV-2 pulmonary and cardiac injury in the vaccinated patients, a field that has until now remained unexplored. We underscored the importance of vaccine immunity to protect heart from inflammatory viral-induced damage, supporting the importance and the efficacy of the vaccination against Sars-CoV-2, and confirmed, once again, that in the absence of vaccination, even previously healthy young people can be at risk of mortality due to severe COVID-19. Vaccine response decreases over time and, as observed in our cases, COVID-19 may contribute to deteriorate a compromised condition due to underlying comorbidities, leading to death. Vaccine boosters are crucial in order to enhance or restore protection.

## Figures and Tables

**Table 1:** Demographic, and clinical course of vaccinated patients.

N°	Age/ Sex	Time since Second Vaccine Dose	Comorbidities	Symptoms Before Hospital Stay	SARS-CoV2 positivity Before Hospitalization	Hospital Stay (Days)	Pulmonary Imaging	Oxygen/Intensive Care	Certified Cause of Death
1	78 F	195 days (6.5 months)	Patient bedridden for 10 years for Parkinson; implant for deep brain stimulation (DBS)	Fever and pneumonia	Not known	Deceased at the ER	/	/	Acute respiratory distress syndrome due to COVID-19
2	81 M	270 days (9 months)	Chronic ischemic cardiomyopathy; Hospitalized for malignancy	Pneumonia during hospitalization in the oncological unit	Negative *	11 ^**1**^	CT: bilateral multifocal ground-glass opacities	Oxygen therapy	Myocardial infarction, respiratory failure due to bacterial bronchopneumonia and abdominal fibromatosis
3	60 F	188 days (6.5 months)	Hypertension; Paroxysmal AFib; Hypotiroidism; rheumatoid arthritis; incarcerated umbilical hernia; active cancer in the past 5 years	Syncopal episode	11 days	Deceased at the ER	CT: bilateral basal ground-glass opacities	Orotracheal intubation	Heart failure due to auricle thrombosis and small bowel ischemia
4	66 M	250 days (8.5 months)	ALD, hepatic cirrhosis, multiple cysts splenomegaly; multi-infarct leukoencephalopathy	/	Negative *	15	CT: Superior right lobe areas of ground-glass opacities	Refused	Respiratory failure due to bacterial pneumonia, cirrhotic cardiomyopathy and encephalopathy
5	75 M	298 days (10 months)	COPD; epilepsy; rheumatoid arthritis; cerebral vasculopathy; diabetes	3 days fever	3 days	23	CT: bilateral multifocal crazy paving	Refused	Rheumatoid arthritis related organizing pneumonia

AFib = Atrial Fibrillation; ALD = Alcoholic Liver Disease. ER = emergency room. 1 = Referred to hospitalization in COVID-19 Hospital Unit; * positivization during hospital stay.

**Table 2:** Demographic, and clinical course of unvaccinated patients.

N°	Age/Sex	Comorbidities	Symptoms Before Hospital Stay	SARS-CoV2 Positivity Before Hospitalization	Hospital Stay (Days)	Pulmonary Imaging	Oxygen/Intensive Care	Certified Cause of Death
1	67 F	Emphysema	4 days general malaise; hypotension with syncopal episodes	Not known	Deceased at the emergency room	CT: bilateral and diffuse crazy paving pattern	/	Myocarditis and acute respiratory distress syndrome due to COVID-19
2	53 F	/	4 days progressive dyspnea	15 days	Deceased at the emergency room	/	/	Myocarditis, acute respiratory distress syndrome, and haemorrhagic infarction due to COVID-19
3	51 M	ALD; atherosclerosis	dyspnea	Not known	9	RX: bilateral consolidations involving the majority of both lungs	Oxygen therapy with NIV; orotracheal intubation;	G.I. haemorrhage and lobar pneumonia
4	49 M	Obesity (BMI ≥ 30)	3 days progressive dyspnea	Not known	18	CT: bilateral and diffuse crazy paving pattern	Oxygen therapy with VMK 50%; NIV; orotracheal intubation;	Acute respiratory distress syndrome due to COVID-19
5	45 F	Obesity (BMI ≥ 30)	Fever; nausea	9 days	4	CT: bilateral and multifocal ground-glass opacities	Orotracheal intubation	Acute respiratory distress syndrome due to COVID-19

ALD = Alcoholic Liver Disease; NIV = not invasive ventilation; VMK = mechanical ventilation.

**Table 3:** Postmortem SARS-CoV-2 RNA detection.

	Unvaccinated Patients	Vaccinated Patients	
Sample			*p* Value
Bronchi (median/range)	23.37 (17.78–34.42)	31.24 (26.35–34.49)	0.1019
Lungs (median/range)	21.49 (14.51–32.59)	30.54 (18.10–37.69)	0.0016 *
Myocardium (median/range)	33.93 (26.03–39.63)	31.21 (27.12–35.30)	0.6821

Ct values were showed. * Significant value (*p* < 0.005).

**Table 4:** Macroscopic lung findings.

N°	Sex	Weight (g)	Edema	Congestion	Right Lung Consolidation			Left Lung Consolidation		Bacterial Bronchopneumonia/ Pneumonia	Fibrosis	PA	PE
					Upper Lobe	Medium Lobe	Lower Lobe	Upper Lobe	Lower Lobe				
**UNVACCINATED PATIENTS**													
1	F	Right 576 Left 385 Total 961	+	+	/	Diffuse	Diffuse	Diffuse	Focal	/	Bilateral, subpleural	/	/
2	F	Right 1200 * Left 638 Total 1838 *	+	/	Diffuse	Diffuse	Diffuse	Diffuse	Diffuse	/	Bilateral	+	/
3	M	Right 1027 * Left 1112 * Total 2139 *	+	+	Diffuse	Diffuse	Diffuse	Diffuse	Diffuse	Bilateral	Supleural		/
4	M	Right 939 * Left 854 * Total 1793 *	/	+	Diffuse	Diffuse	Diffuse	Diffuse	Diffuse	/	Bilateral	/	/
5	F	Right 882 * Left 720 * Total 1602 *	+	+	Diffuse	Diffuse	Diffuse	Diffuse	Diffuse	/	Bilateral	/	/
**VACCINATED PATIENTS**													
1	F	Right 563 Left 583 Total 1146	+	/	Diffuse	Diffuse	Diffuse	Diffuse	Diffuse	/	Bilateral	/	/
2	M	Right 702 Left 662 Total 1364 *	/	+	/	/	Focal	/	Diffuse	Lower lobes	Lower lobes	/	+
3	F	Right 510 Left 519 Total 1029	+	+	Focal	Focal	Diffuse	/	Diffuse	Right lung	Lower left lung	/	/
4	M	Right 1080 * Left 662 Total 1742 *	+	+	Diffuse	Diffuse	Diffuse	/	/	Right lung	Focal	/	/
5	M	Right 620 Left 491 Total 1111	/	+	Focal	/	Diffuse	Diffuse	Focal	Lower left lobe	Bilateral focal	/	/

* Greater than the upper limit for normal lung weight; PA = Pulmonary artery thrombosis; PE = Pulmonary embolism; (+) = Present; (/) = Absent.

**Table 5:** Macroscopic heart findings.

N°	Sex	Weight (g)	Cardiac Hypertrophy	Dilatation of the Chambers	LV Wall Thickness (mm)	RV Wall Thickness (mm)	Consistency	Septum Thickness (mm)	Obstructive Modifications
**UNVACCINATED PATIENTS**									
1	F	436	/	/	13	3	Flabby	10	Mild aortic atherosclerosis
2	F	470	Concentric hypertrophy	/	**18**	4	Firm	**25**	Moderate aortic atherosclerosis
3	M	424	Eccentric hypertrophy	LV dilatation	12	3	Flabby	11	Mild aortic atherosclerosis
4	M	797	Concentric hypertrophy	/	12	3	Firm	14	Mild aortic atherosclerosis
5	F	396	Eccentric hypertrophy	Biventricular dilatation	12	3	Flabby	12	/
**VACCINATED PATIENTS**									
1	F	450	Eccentric hypertrophy	Biventricular dilatation	12	4	Flabby	12	Moderate aortic atherosclerosis; coronary stenosis above 50%
2	M	355	Eccentric hypertrophy	Biventricular dilatation	10	3	Flabby	15	Aortic and mitral valve stenosis; moderate aortic atherosclerosis
3	F	427	/	Biventricular dilatation	8	3	Flabby	10	Mild aortic atherosclerosis
4	M	470	Concentric hypertrophy	/	**18**	5	Firm	**20**	Moderate aortic atherosclerosis; coronary stenosis of one
5	M	332	/	Biventricular dilatation	12	4	Flabby	12	Mild aortic atherosclerosis

Abnormal values are in bold. Pathological parameters: Left Ventricular (LV) wall, >15 mm; Right Ventricular (RV) wall, >5 mm; Septum, >15 mm. (/) = Absent.

## Data Availability

All relevant data are within the manuscript.

## Supplementary Materials

The following supporting information can be downloaded at: https://www.mdpi.com/article/10.3390/biomedicines11020551/s1, Supplementary Figure S1: Distribution of total lung weight in vaccinated and unvaccinated patients; Figure S2: Inflammatory cells in lung tissue from vaccinated and unvaccinated patients; Figure S3: Distribution of heart weight in vaccinated and unvaccinated patients; Figure S4: Inflammatory cells immune-characterization in myocardial tissue from unvaccinated elderly COVID-19 deceased patients; Table S1: Post-mortem PCR detection of SARS-CoV-2 in different organs; Table S2: Brain histopathological findings.
