# Supplementary material for: Autopsies Revealed Pathological Features of COVID-19 in Unvaccinated vs. Vaccinated Patients
Source: Biomedicines. 2023 Feb 14;11(2):551. doi: 10.3390/biomedicines11020551 (PMC9953314; doi:10.3390/biomedicines11020551)
Supplement: Supplementary file 1 [file biomedicines-11-00551-s001.zip › Suppl MATERIALS merged.pdf]

“Autopsies revealed pathological features of COVID-19 in unvaccinated vs vaccinated patients”

Table S1

Post-mortem PCR detection of SARS-CoV-2 in different organs.

	Sample								
	Nasopharyngeal swab	Right Broncus	Left Broncus	Lung Right lobe	Lung Left lobe	Myocardium	Brain	Liver	Kidney
**Vaccinated Patients**									
n.1	Gray	Gray	Gray	Red	Red	Red	Green	Gray	Gray
n. 2	Red	Red	Red	Green	Red	Red	Gray	Gray	Red
n. 3	Gray	Red	Red	Red	Red	Gray	Gray	Gray	Gray
n. 4	Red	Red	Red	Red	Red	Green	Red	Gray	Red
n. 5	Red	Red	Red	Red	Red	Green	Green	Green	Green
**Unaccinated Patients**									
n.1	Red	Red	Red	Red	Red	Red	Green	Gray	Gray
n. 2	Red	Red	Red	Red	Red	Red	Green	Gray	Gray
n. 3	Red	Red	Red	Red	Red	Red	Red	Red	Gray
n. 4	Red	Red	Green	Red	Red	Green	Gray	Green	Green
n. 5	Red	Red	Red	Red	Red	Red	Gray	Green	Red

Red = positive,; green = negative,; gray = test not performed

“Autopsies revealed pathological features of COVID-19 in unvaccinated vs vaccinated patients”

Fig S1

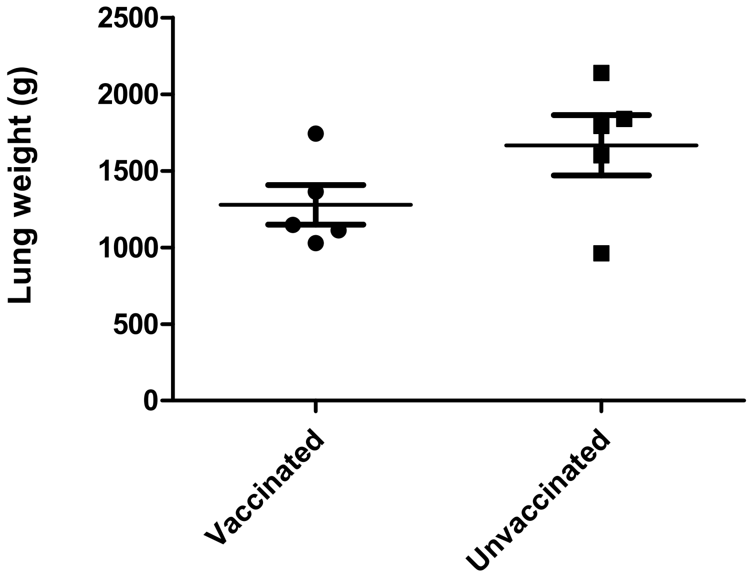

Figure S1 Distribution of total lung weight in vaccinated and unvaccinated patients. Horizontal bars indicate the median values

“Autopsies revealed pathological features of COVID-19 in unvaccinated vs vaccinated patients”

Fig S2

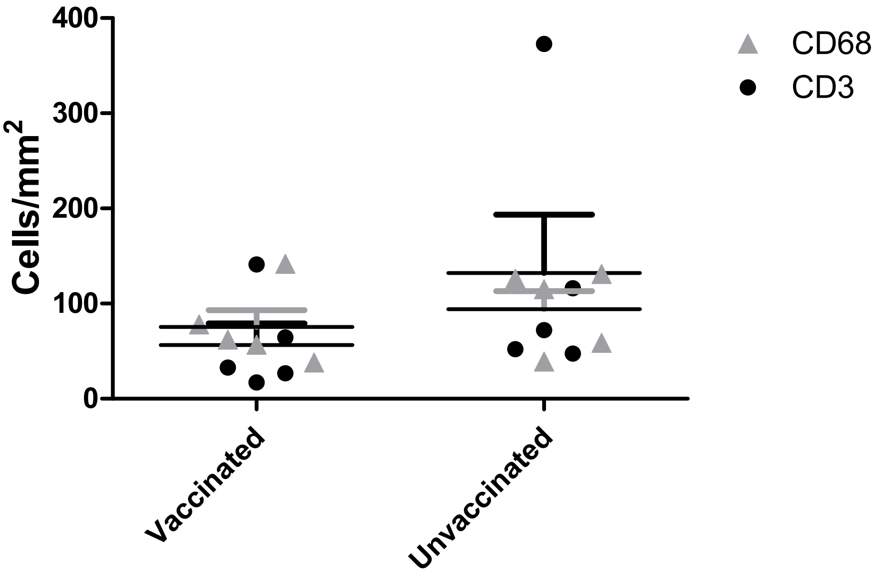

Figure S2. Inflammatory cells in lung tissue from vaccinated and unvaccinated patients. Each point represents the mean number of CD68+ and CD3+ cells per mm2, for each patient.

“Autopsies revealed pathological features of COVID-19 in unvaccinated vs vaccinated patients”

Fig S3

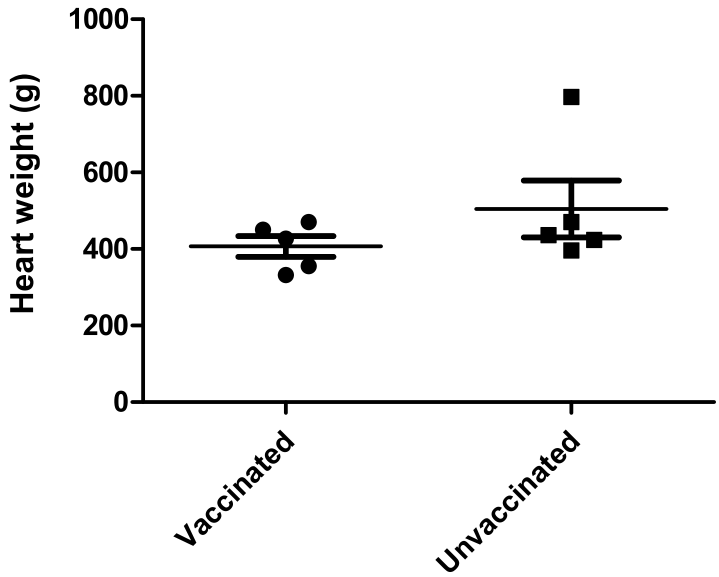

Figure S3 Distribution of heart weight in vaccinated and unvaccinated patients. Horizontal bars indicate the median values

“Autopsies revealed pathological features of COVID-19 in unvaccinated vs vaccinated patients”

Fig S4

A

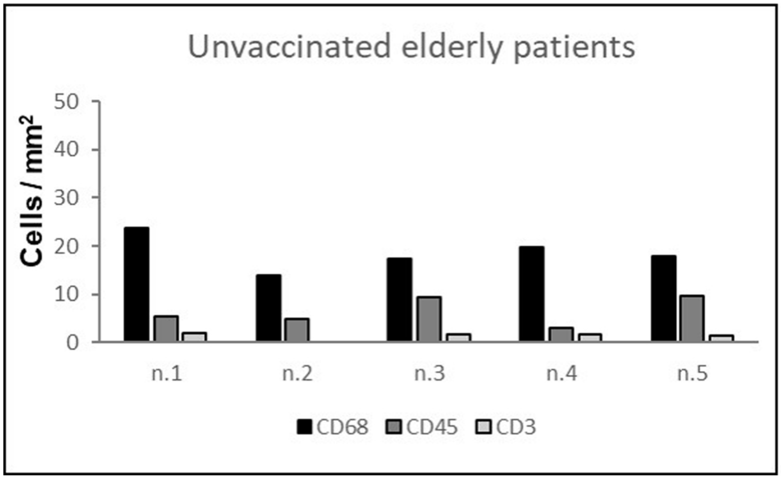

B

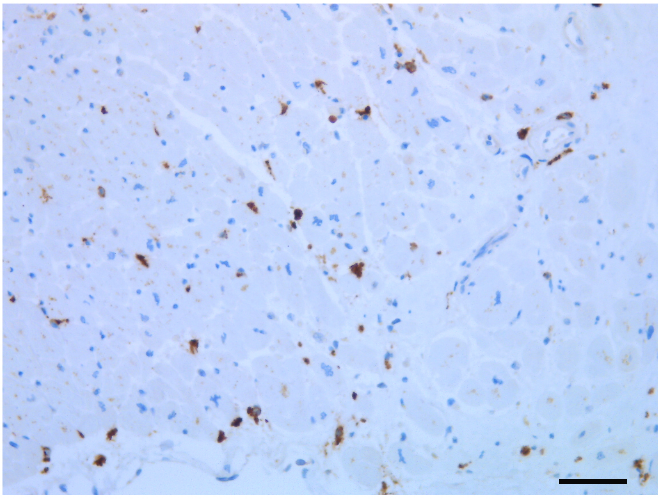

Figure S4 Inflammatory cells immune-characterization in myocardial tissue from unvaccinated elderly COVID-19 deceased patients. (A) Quantification of the number of extravascular CD68+ macrophages, CD45+ leukocytes, and CD3+ T lymphocytes. For each case (n.1-n.5) the mean number of cells per mm2 is reported. (B) Representative photomicrograph of anti-CD68 immunohistochemistry. The presence of numerous labelled macrophages is visible.
Scale bars: B = 28 µm.

“Autopsies revealed pathological features of COVID-19 in unvaccinated vs vaccinated patients”

Table S2. Brain histopathological findings.

N°	Pre-existing medical conditions affecting brain	RT-PCR results on brain tissue	Neuropathological findings
**UNVACCINATED PATIENTS**			
1	None	Negative	Axonal vacuolization, degeneration of neurons, dilatation and congestion of vessels with perivascular inflammatory infiltrate mainly consisting in T lymphocytes (CD3+), cytotoxic (CD8+), focal perivascular hemorrhagic extravasations
2	None	Negative	Widespread presence of corpora amylacea in the subependymal site and mostly involving the brainstem associated to chronic inflammation substained by CD3 T lymphocytes, CD8 positive , mostly perivascular. Focal perivascular hemorrhagic extravasations at the cortical and thalamic level where it is also present rarefaction of the parenchyma. Cerebral artery dilatation with fibrointimal hyperplasia, wall calcifications and hemorrhages
3	Hepatic encephalopaty	Positive	Areas of petechial hemorrhages with vascular endothelial proliferation, spongiosis and proliferation of astrocytes characterized by nuclear pallor, swelling, and chromatin margination; microglial activation is associated with the formation of microglial nodules mixed with debris necrotic and inflammatory cells (neutrophils and macrophages)
**VACCINATED PATIENTS**			
1	Parkinson’s disease treatment with “deep brain stimulation”	Negative	Brain Atrophy
4	Hepatic encephalopaty	Positive	Microglial activation, swollen astrocytes with pale nuclei, eosinophilic nucleoli, scanty cytoplasm, perivascular blood extravasations
5	Epilepsy, cerebral vasculopathy	Negative	Perivascular microhemorrages, microglial nodules and astroglial activaction due to ischemic hypoxic damage with small vessels damage and hyaline arteriolosclerosis
